# Assessing the dependence of brain activity on individual single-nucleotide variability of genetic markers of major depressive disorder using principal component analysis

**DOI:** 10.18699/vjgb-25-117

**Published:** 2025-12

**Authors:** K.A. Zorina, A.A. Kriveckiy, V.S. Karmanov, A.N. Savostyanov

**Affiliations:** Novosibirsk State University, Novosibirsk, Russia; Novosibirsk State Technical University, Novosibirsk, Russia; Novosibirsk State Technical University, Novosibirsk, Russia; Novosibirsk State University, Novosibirsk, Russia Institute of Cytology and Genetics of the Siberian Branch of the Russian Academy of Sciences, Novosibirsk, Russia Scientific Research Institute of Neurosciences and Medicine, Novosibirsk, Russia

**Keywords:** stop-signal paradigm, EEG, event related potentials, single nucleotide polymorphisms (SNPs), major depressive disorder, principal component analysis, regression analysis, стоп-сигнал парадигма, ЭЭГ, вызванные потенциалы, однонуклеотидные полиморфизмы, большое депрессивное расстройство, метод главных компонент, регрессионный анализ

## Abstract

Major depressive disorder (MDD) is one of the most widespread mental illnesses, which necessitates the search for factors of increased predisposition to this disorder. Single nucleotide polymorphisms in genes of the brain’s neurotransmitter systems are often considered as molecular genetic markers of MDD. Indicators of individual single nucleotide variability in neurotransmitter genes are used to assess the risk of MDD before its symptomatology at the behavioral level. However, the predictive capabilities of analyzing genomic variations to assess the risk of depression are not yet sufficiently reliable and are complemented by behavioral and neurophysiological information about patients. Neurophysiological markers of MDD provide the most reliable estimates of the severity of pathological symptoms, but they reflect a person’s state at the time of examination, and not a predisposition to the occurrence of this pathological state and do not allow assessing the risk of its appearance in the future. Major depressive disorder is often accompanied by abnormalities in a person’s ability to control motor responses, including the ability to voluntary suppress inappropriate behavior. The “stop-signal paradigm” (SSP) is an experimental method for assessing the functional balance between the inhibitory and activation systems of the brain during targeted movements. Combined with EEG recording, this experimental method allows for the consideration of not only participants’ behavioral characteristics, such as speed or accuracy of responses, but also the brain’s neurophysiological features associated with behavior control. The objective of this study was to evaluate the relationship between EEG responses in the stop-signal paradigm and individual single nucleotide variability in candidate genes for MDD detection. Dimensionality in the original genetic and neurophysiological experimental data was reduced by principal component analysis (PCA) to subsequently detect an association between EEG response components recorded during the control of random motor responses and single nucleotide variations in genes, the variability of which is asso-ciated with MDD risk. Variability in these genes has been shown to be associated with the amplitude of brain responses under the conditions of test subjects using the PCA method. The results obtained can be used to develop systems for the early diagnosis of depression, identify individual patterns of impairment in the brain, select methods for correcting the disease and control the effectiveness of therapy.

## Abstract (ru)

Большое депрессивное расстройство (БДР) относится к наиболее широко распространенным психическим заболеваниям, что обусловливает необходимость поиска факторов повышенной предрасположенности к этому нарушению. В качестве молекулярно-генетических маркеров БДР часто рассматривают однонуклеотидные полиморфизмы генов нейромедиаторных систем мозга. Показатели индивидуальной однонуклеотидной вариабельности в генах нейромедиаторов применяются для оценки риска появления БДР до проявления его симптоматики на поведенческом уровне. Однако прогностические возможности анализа геномных вариаций для оценки риска депрессии до настоящего времени недостаточно надежны и дополняются поведенческой и нейрофизиологической информацией о пациентах. Нейрофизиологические маркеры БДР дают наиболее надежные оценки выра- женности патологической симптоматики, но они отражают состояние человека в момент обследования, а не предрасположенность к возникновению этого патологического состояния и не позволяют выполнить оценку риска его появления в будущем. Большое депрессивное расстройство часто сопровождается отклонениями в способности человека контролировать двигательные реакции, включая возможность произвольно подавлять неадекватное поведение. «Стоп-сигнал парадигма» (ССП) – экспериментальный метод для оценки функционального баланса между тормозными и активационными системами головного мозга в условиях выполнения целенаправленных движений. Объединенный с регистрацией ЭЭГ, этот экспериментальный метод позволяет учитывать не только поведенческие характеристики участников, такие как скорость или точность ответов, но и нейрофизиологические особенности головного мозга, ассоциированные с контролем над поведением. Цель настоящего исследования заключалась в оценке зависимости между особенностями ЭЭГ реакций в условиях парадигмы стоп-сигнал и индивидуальной однонуклеотидной вариабельностью в генах-кандидатах для выявления БДР. Размерность в исходных генетических и нейрофизиологических экспериментальных данных была снижена при помощи анализа главных компонент (РСA) для последующего выявления ассоциации между компонентами ЭЭГ реакций, регистрируемыми в условиях контроля произвольных двигательных реакций, и однонуклеотидными вариациями в генах, изменчивость которых ассоциирована с риском БДР. Установлено, что изменчивость в этих генах ассоциирована с амплитудными показателями мозговых ответов в условиях тестирования испытуемых методом ССП. Полученные результаты могут быть использованы для разработки систем ранней диагностики депрессии, выявления индивидуальных паттернов нарушения в работе головного мозга, подбора методов коррекции заболевания и контроля над эффективностью терапии.

## Introduction

Major depressive disorder (MDD), also known as clinical depression, is a psychiatric disorder characterized by symptoms including depressed mood, loss of interest or pleasure in previously enjoyable activities, fatigue or loss of energy, alterations in sleep and appetite, difficulties with concentration and memory, as well as feelings of guilt and low self-esteem (DSM-5, 2013). MDD ranks among the most prevalent psychiatric disorders (Wong, Licinio, 2001). Susceptibility to various forms of depressive disorders is known to depend on both genetic factors and individual life experiences, particularly during the period preceding the onset of MDD symptoms (Cross-Disorder Group, 2013; Northoff, 2013; Haase, Brown, 2015; Ivanov et al., 2019; Whitney et al., 2019). For many years, the monoamine theory of depression was considered the most plausible, and allelic polymorphisms in genes encoding components of the brain’s monoaminergic neurotransmitter systems have frequently been investigated as molecular markers of depression susceptibility (Willner et al., 2013). However, attempts to predict depression risk based solely on genetic data have generally proven unsatisfactory (Duncan et al., 2014; Halldorsdottir, Binder, 2017), as depression is a multifactorial disorder arising from the interplay of multiple genetic and environmental factors (Ivanov et al., 2019; Wang et al., 2025). Consequently, the identification of reliable biomarkers for depression necessitates the concurrent use of not only genetic but also neurophysiological indicators reflecting the functional state of the human brain.

Neurophysiological markers of depression may include the amplitude and latency of event-related potentials (ERPs) derived from electroencephalography (EEG) (Stone et al., 2025). It is well established that depression is frequently associated with impairments in inhibitory control, manifesting at both behavioral and neurophysiological levels (Shetty et al., 2025). An example of a method used to assess individual capacity for behavioral self-control is the stop-signal paradigm (SSP) (Band et al., 2003). This experimental paradigm provides an objective measure of the functional balance between brain activation systems that govern goal-directed actions and inhibitory systems responsible for suppressing inappropriate behavior.

A major challenge in the comprehensive investigation of depression lies in the need to account for a large number of variables, the interrelationships of which are not initially evident to the researcher. This challenge can be addressed through the application of dimensionality reduction techniques designed to uncover latent dependencies among factors. In particular, principal component analysis (PCA) is widely employed to reduce the dimensionality of original datasets and to identify the most informative features (Gewers et al., 2021). PCA transforms the original variables into a lowerdimensional space, thereby reducing the number of parameters under analysis and mitigating redundancy inherent in highdimensional data (Subasi, Gursoy, 2010).

The aim of the present study was to investigate the association between neurophysiological measures recorded during the stop-signal paradigm and individual single-nucleotide variability in genes linked to an elevated risk of depression

In this work, we analyzed genetic and neurophysiological data obtained from the publicly available ICBrainDB, developed by researchers at the Institute of Cytology and Genetics, Siberian Branch of the Russian Academy of Sciences (ICG SB RAS), and the Institute of Neuroscience and Medicine, and hosted on the ICG SB RAS website (Ivanov et al., 2022). Candidate genes for MDD had been previously selected through a bioinformatic analysis of scientific publications retrieved from open-access databases containing information on depressive spectrum disorders diagnosed in the studied individuals (Ivanov et al., 2019).

## Materials and methods

Participant sample. The sample comprised 212 individuals for whom both genomic and EEG data were analyzed. Among them, 47 participants residing in Novosibirsk had a clinically diagnosed major depressive disorder, while 165 participants had no diagnosed depression; of these, 67 resided in Novosibirsk, 50 in Yakutsk, and 48 in Khandyga, Sakha Republic

Experimental design. Participants performed a series of tasks in a stop-signal paradigm modified by A.N. Savostyanov and colleagues (2009). During the task, one of two visual stimuli was presented on the screen; upon the appearance of the target stimulus, participants were required to press a button on the keyboard. On a subset of trials, a stop-signal appeared shortly after the target stimulus, instructing the participant to abort the already initiated motor response. Across the experiment, each participant completed 135 trials, 35 of which included a stop-signal. EEG was recorded using a 128-channel NVX-132 amplifier. Electrodes were positioned according to the international 10-5 system, with AFz serving as the ground electrode and Cz as the reference. The signal bandwidth was set between 0.3 and 100 Hz, and the sampling rate was 1,000 Hz.

EEG signal processing. Raw EEG recordings contained non-neural noise, including ocular movement artifacts, facial muscle activity, cardiac electrical activity, and vascular artifacts. All non-neural artifacts were removed using independent component analysis (ICA), implemented in the EEGLAB toolbox (Delorme, Makeig, 2004). ICA is a computational algorithm that decomposes multichannel data into statistically independent components. In contrast, PCA identifies components characterized by high mutual dependence.

From the preprocessed EEG data, two types of epochs were extracted: go-epochs (intervals of brain activity time-locked to the participant’s button press following the target visual stimulus) and stop-epochs (intervals corresponding to successful inhibition of the motor response after stop-signal presentation). Epoching for go-trials was performed relative to the onset of the target stimulus, whereas for stop-trials it was aligned to the onset of the stop-signal. Within go-epochs, two distinct EEG peaks were identified: a premotor peak (400–600 ms poststimulus) and a postmotor peak (700–800 ms post-stimulus). The premotor peak reflects brain activity associated with motor preparation, whereas the postmotor peak corresponds to neural processes occurring during movement execution

Genetic data. Genetic material, collected as either whole blood or buccal epithelial cells, was obtained from all participants. Targeted sequencing of 164 genes was performed using this material. These genes were selected based on prior reconstruction and analysis of a gene network associated with susceptibility to MDD (Ivanov et al., 2019). Targeted sequencing libraries were prepared for these 164 genes, and high-coverage next-generation sequencing (NGS) was conducted for all participants. For each allele of every gene in the list, a binary variability index was assigned for each participant relative to the reference genome (Ivanov et al., 2022). If a participant’s allele sequence matched the reference genome exactly, the variability index was set to 0; if one or more nucleotide substitutions were present, the index was set to 1 (regardless of the number of substitutions within the allele). Across all participants, 799 single-nucleotide polymorphisms were identified in 121 of the 164 sequenced genes. No nucleotide substitutions were detected in any participant for the remaining 43 genes. Thus, the total number of input genetic parameters was 242 (121 genes × 2 alleles per gene).

## Results

As previously stated, the aim of this study was to assess the association between EEG responses recorded during the stopsignal paradigm and individual single-nucleotide variability in candidate genes linked to MDD risk. To achieve this objective, a multi-stage analysis of the experimental data was conducted, and the results are presented below

**Task 1. Identification of MDD candidate genes exhibiting significant associations between single-nucleotide variability and EEG measures**

To address Task 1, a series of linear models was constructed, wherein each EEG parameter served as a dependent variable and the binary indicator of the presence or absence of singlenucleotide variants (SNVs) in a specific gene served as the independent variable. The term “linear model series” refers to separate linear regression analyses performed for each unique pair of “EEG parameter – single-nucleotide variability” (Table 1). Given 144 EEG parameters and 242 genetic parameters, the initial number of parameter pairs subjected to linear regression totaled 34,848. An individual linear regression model was formulated as follows:

**Formula. 1.**
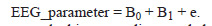
Formula. 1.

Here, В1 represents the binary predictor coded as 0 (no nucleotide substitution in the allele) or 1 (at least one substitution present).

**Table 1.**
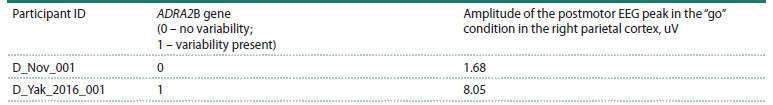
Example of a parameter pair used in linear regression analysis. The first parameter is individual variability in the ADRA2B gene; the second is the amplitude of the premotor ERP peak in the right parietal cortex

The dependent variable was a quantitative EEG measure, while the predictor was the binary indicator of nucleotide substitution presence in a given gene allele. If at least one substitution was present in one allele, the binary indicator was assigned a value of 1. The two alleles of the same gene were treated as two distinct binary predictors. This approach enabled testing whether single-nucleotide variability in each candidate gene was associated with alterations in a given EEG parameter.

In addressing Task 1, multiple comparisons were corrected using the Benjamini–Hochberg procedure (False discovery rate, FDR) to control the expected proportion of false rejections of the null hypothesis (Benjamini, Hochberg, 1995). The FDR method is more statistically powerful than the Bonferroni correction and is particularly advantageous when the number of tested hypotheses is large or when minimizing false positives is prioritized over strict per-hypothesis control of Type I error

Associations were tested between all 144 EEG measures and variability in each of the 121 genes in which at least one SNV was detected in at least one participant. This analysis revealed statistically significant associations (FDR-corrected significance threshold q < 0.05) for only five genes – ADRA2B, TF, HCRTR2, WFS1, and PENK – and four EEG measures recorded during go-epochs in the medial frontal, right parietal, left parietal, and combined occipital cortical regions (Table 2). Notably, significant associations for three genes (ADRA2B, TF, HCRTR2) were observed across three cortical regions (right parietal, left parietal, and occipital), whereas for the remaining two genes (WFS1 and PENK), significant associations were confined to the medial frontal cortex. These five genes were subsequently included in further analyses

**Table 2.**
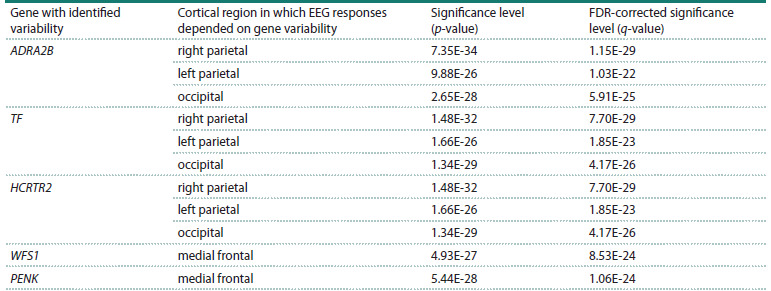
Results of the association analysis between the amplitude of the postmotor ERP peak in go-episodes of the stop-signal paradigm and binary variability in MDD candidate genes

Table 2 summarizes the linear regression results linking EEG measures to polymorphisms in MDD candidate genes. It lists 11 most significant “gene–EEG measure” pairs with the lowest FDR-corrected p-values (q-values), along with their uncorrected p-values. All reported associations are significant at FDR < 0.05.

The average frequency of single-nucleotide variants for each of the five selected genes across the entire participant sample is presented in Table 3. The prevalence of variant carriers for these genes ranged from approximately one-third to two-thirds of participants, ensuring sufficient variability for robust statistical analysis.

**Table 3.**
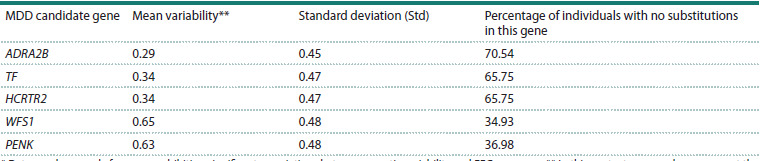
Mean number of single-nucleotide variants in selected MDD* candidate genes Data are shown only for genes exhibiting significant associations between genetic variability and EEG measures. ** In this context, mean values represent the proportion of participants in the sample who carried at least one nucleotide substitution in the respective gene.

**Task 2. Dimensionality reduction of neurophysiological data using principal component analysis**

In addressing Task 2, PCA with prior feature standardization was applied to reduce the dimensionality of the EEG dataset (Rokhlin et al., 2010). From the original set of 144 EEG variables, 15 principal components were extracted. The Figure demonstrates that these 15 components collectively account for approximately 80 % of the total variance in the original EEG parameters, thereby capturing the majority of inter-individual variability.

**Fig. 1.**
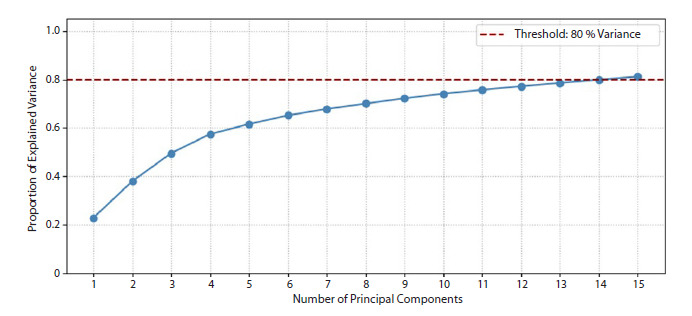
Cumulative variance explained by principal component analysis of EEG data. The red dashed line indicates the 80 % variance threshold.

**Task 3. Assessment of the influence of variability in MDD candidate genes on integrated measures of brain activity derived from PCA**

In Task 3, for each of the five selected genes showing statistically significant associations with specific EEG measures (Table 2), a regression analysis was performed between the principal components (PCs) and the binary indicators of polymorphism presence. Unlike in Task 1, where regression was conducted on individual EEG parameters, here the analysis was performed on integrated composite measures (the principal components) that collectively explain 80 % of the total inter-individual variance in the EEG data (see the Figure).

Among the 15 PCA-derived components of brain activity, only the third principal component (PC3) exhibited a statistically significant association with genetic variability in the MDD candidate genes. This finding is summarized in Table 4, which presents the results of statistical significance testing for the effects of genetic variability in the five candidate genes on the three most informative PCA components.

**Table 4.**
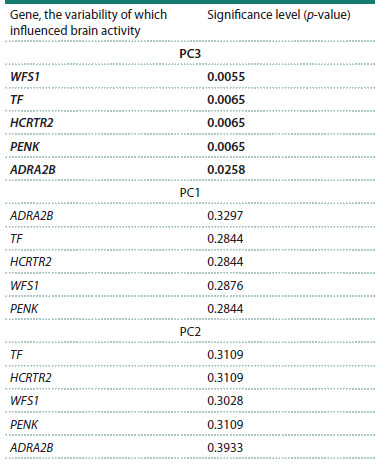
Results of linear regression between the first three EEG principal components (PC1–PC3) and variability in the five MDD candidate genes* * Results are ordered by the significance level of the linear regression

To provide a neurophysiological interpretation of the observed associations, factor loadings for the third principal component (PC3) were computed for each of the original EEG measures. In the context of PCA, a factor loading represents the correlation coefficient between an original variable and a principal component, indicating the strength and direction of their association. The factor loadings of the original EEG measures for PC3 are presented in Table 5. As evident from these results, PC3 is most strongly associated with brain activity in occipito-parietal cortical regions and, to a somewhat lesser extent, with frontal cortical activity. This cortical topography is characteristic of functional processes involved in attentional control during visual stimulus recognition. Furthermore, it is apparent that both premotor and postmotor ERP peaks-across both go- and stop-episodes contributed most substantially to this component.

**Table 5.**
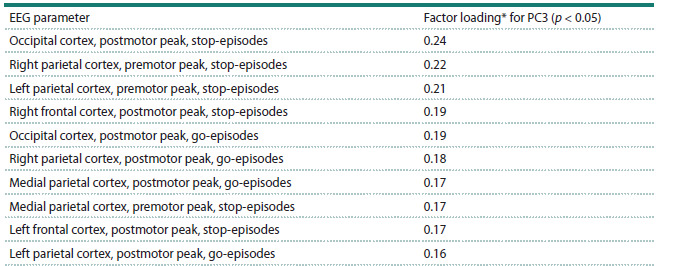
Factor loadings of original brain activity measures for PC3 * Factor loading denotes the correlation coefficient between an EEG measure and the integrated score of PC3

**Task 4. Prediction of candidate gene variability based on composite EEG measures (solving the inverse problem)**

To address this task, logistic regression with L1 regularization (Flach, 2016) was employed to predict the presence or absence of single-nucleotide variants in MDD candidate genes using the first 15 EEG-derived principal components (PC1–PC15). Unlike linear regression, which models continuous dependent variables, logistic regression is designed for binary outcomes.

In our case, the logistic model aimed to estimate the probability of genetic variability in MDD candidate genes based solely on EEG-derived features, thus constituting the inverse problem. The input features consisted of the first 15 principal components extracted from the original EEG parameter space, while the target variables were binary indicators of deviation from the human reference genome in the five genes previously shown to exhibit significant associations with EEG components: ADRA2B, WFS1, PENK, TF, and HCRTR2. Model performance was evaluated using the area under the receiver operating characteristic curve (AUC), computed via 5-fold stratified cross-validation. The accuracy estimates (AUC values) and their standard deviations across the five cross-validation folds are presented in Table 6. As shown in Table 6, prediction accuracy for binary genetic variability in three of the five candidate genes ranged from 0.73 to 0.78, with standard deviations between 0.13 and 0.18. These results indicate that the presence of binary variability in MDD candidate genes can be predicted from EEG data recorded during the stop-signal paradigm with 70–80 % accuracy, thereby providing convergent evidence for a robust link between genetic susceptibility and neurophysiological phenotypes

**Table 6.**
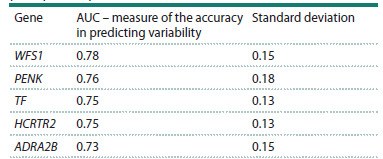
Results of logistic regression for predicting the presence of mutations using the 15 EEG principal components

Thus, the sequential accomplishment of the four objectives of our study enabled us to: (1) identify a list of candidate genes for MDD, the variability of which is associated with measures of brain activity during behavioral control; (2) determine composite characteristics of brain activity accounting for 80 % of the variance in EEG data; (3) identify an integrated measure of brain activity most robustly associated with single-nucleotide variability in MDD candidate genes; and (4) solve the inverse problem by predicting variability in MDD candidate genes based on EEG-derived measures.

## Discussion

A fundamental challenge in identifying candidate genes for most psychiatric disorders is that the behavioral effects of single-nucleotide variations in any individual selected gene are relatively weak (Duncan et al., 2014). Depression exemplifies a disorder for which no direct and unambiguous associations with specific g‑enes have been established (Halldorsdottir, Binder, 2017). This suggests that the genetic underpinnings of depression are highly heterogeneous across individuals and cannot be reduced to a small set of genes and their mutation

This has motivated a shift in focus from analyzing the contribution of individual genes or mutations toward investigating interconnected complexes of genes, their protein products, and metabolites. Such gene complexes are referred to as “gene networks” (Kolchanov et al., 2013). A gene network may encompass dozens to hundreds of genes, along with the multitude of proteins and metabolites they encode. Previously, using bioinformatic approaches, fragments of a gene network implicated in susceptibility to major depressive disorder (MDD) were reconstructed (Ivanov et al., 2019). In the same study, a comprehensive dataset was assembled, integrating psychometric, neurophysiological, and genetic data reflecting the analysis of SNPs across 164 genetic loci incorporated into the depression-related gene network (Ivanov et al., 2022). The aim of the present study was to identify genes associated not only with psychometric traits but also with neurophysiological characteristics of brain activity, which may likewise be considered as manifestations of depression.

Behavioral control is one of the core cognitive functions in humans, and its impairment constitutes a symptom of numerous neuropsychiatric disorders. In the present study, we analyzed the relationship between parameters of human ERPs and the presence of single-nucleotide variations in candidate genes for MDD within a combined sample comprising both healthy individuals and those diagnosed with depressive disorder. Our results demonstrate that the amplitude of the postmotor positivity in go-trials of the stop-signal paradigm is associated with binary variability in five MDD candidate genes: ADRA2B, TF, HCRTR2, WFS1, and PENK (Table 2)

Associations with genetic variability were observed not only for several localized EEG measures reflecting cortical activity in specific brain regions during brief phases of task performance but also for an integrated measure of global brain activity derived via PCA, which captures more general features of the nervous system’s functional state (Table 4). This integrated brain activity measure significantly influenced by genetic variability reflects the engagement of cortical regions involved in visual signal perception and voluntary attentional control (Table 5). Furthermore, we demonstrated that these integrated EEG measures can serve as predictors of singlenucleotide variability in MDD candidate genes with 70–80 % accuracy when applying logistic regression (Table 6), thereby indicating the feasibility of solving the inverse problem: predicting genetic variability from neurophysiological data.

Additional findings from our prior work indicate that ERP amplitudes during performance of the stop-signal paradigm are positively correlated with the severity of depressive symptoms (Zorina et al., 2025). Thus, a coherent link emerges between specific genes, the variability of which is associated both with depression at the behavioral level and with a neurophysiological marker of elevated depressive symptomatology. Information from Ivanov et al. (2019) further clarifies the biological roles of these genes: (a) ADRA2B encodes the alpha- 2B adrenergic receptor, a member of the G protein-coupled receptor family; (b) TF encodes transferrin; (c) HCRTR2 encodes hypocretin (orexin) receptor type 2; (d) WFS1 encodes wolframin; and (e) PENK encodes the proenkephalin precursor protein. Our new findings indicate that variability in these MDD candidate genes is associated with brain activity parameters reflecting an individual’s capacity for behavioral self-control – a function impaired in MDD – thereby supporting the existence of a composite genetic-neurophysiological marker linked to depression risk.

## Conclusion

The present analysis revealed statistically significant associations between polymorphisms in the ADRA2B, TF, HCRTR2, WFS1, and PENK genes and EEG signal characteristics recorded during performance of the stop-signal paradigm. Principal component analysis effectively reduced data dimensionality and enabled the identification of the most informative indices of integrated brain activity. Logistic regression models demonstrated that EEG-derived parameters can predict, with moderate accuracy, the presence of single-nucleotide substitutions in MDD candidate genes. These results may facilitate the assessment of complex interdependencies between genetic and neurophysiological markers associated with depression.

Limitations. This study did not specifically evaluate differences between clinically diagnosed patients with depression and healthy participants. A more detailed comparison of the identified associations between neurophysiological and molecular biological markers of depression remains an objective for future, more granular analyses currently planned in our ongoing research.

## Conflict of interest

The authors declare no conflict of interest.
